# Supplementary material for: Cross-tissue analysis of blood and brain epigenome-wide association studies in Alzheimer’s disease
Source: Nat Commun. 2022 Aug 18;13:4852. doi: 10.1038/s41467-022-32475-x (PMC9388493; doi:10.1038/s41467-022-32475-x)
Supplement: Supplementary file 3 — Description of Additional Supplementary Files [file 41467_2022_32475_MOESM3_ESM.pdf]

Description of Additional Supplementary Files

File Name: Supplementary Data 1
Description: Demographic characteristics of the subjects.

File Name: Supplementary Data 2
Description: A total of 50 CpGs were significantly associated with AD diagnosis in the meta-analysis of blood samples in ADNI and AIBL datasets at *P*-value $< 10^{-5}$. Inverse-variance weighted fixed-effects meta-analysis models were used to combine cohort-specific results from logistic regression models that included covariate variables age, sex, batch, and immune cell-type proportions. Odds ratios (OR) describe changes in odds of AD (on the multiplicative scale) associated with a one percent increase in methylation beta values (i.e., increase in methylation beta values by 0.01) after adjusting for covariate variables. The CpGs were annotated using Illumina annotations, the GREAT software, and the enhancer regions described in Nasser et al. (2021) study (PMID: 33828297). Direction indicates hypermethylation (+) or hypomethylation (-) in AD samples compared to controls in the ADNI and AIBL datasets. All statistical tests are two-sided.

File Name: Supplementary Data 3
Description: Information on brain samples used in cross-tissue meta-analysis.

File Name: Supplementary Data 4
Description: A total of 97 CpGs were prioritized in cross-tissue analysis. These CpGs reached FDR significance (i.e., FDR < 0.05) in cross-tissue and brain sample meta-analyses, and nominal significance (i.e., *P*-value < 0.05) in blood sample meta-analysis. The brain sample meta-analysis results were obtained from Zhang *et al.* (2020) (PMID: 33257653). In brain and blood sample meta-analyses, inverse-variance weighted regression meta-analysis models were applied to the brain and blood sample datasets separately. In the cross-tissue meta-analysis, Stouffer's method was used to combine weighted z-scores (transformed from *P*-values) in all six datasets, where the weights were specified based on the square root of the total number of subjects in each dataset. Because DMRs identified by comb-p vary by datasets, so they cannot be meta-analyzed, we used coMethDMR to compute *P*-values for DMRs at a common set of genomic regions in different datasets. Direction indicates hypermethylation (+) or hypomethylation (-) in AD samples compared to controls in the individual datasets. Enhancer annotations were obtained from Nasser et al. (2021) (PMID: 33828297). All statistical tests are two-sided.

File Name: Supplementary Data 5
Description: A total of 8 CpGs and 1 CpG were significant in the cross-tissue analysis of DNAm differences in blood samples and those from the temporal gyrus (TG) and entorhinal cortex brain regions, respectively. The brain sample results were obtained from Supplementary Data 3 and 5 in Smith et al. (2021) (PMID: 34112773). Stouffer's method was used to combine weighted z-scores (transformed from *P*-values) in all six datasets, where the weights were specified based on the square root of the total number of subjects in each dataset. Direction indicates hypermethylation (+) or hypomethylation (-) in AD samples compared to controls in the individual datasets. All statistical tests are two-sided.

File Name: Supplementary Data 6
Description: Significant associations between AD-associated CpGs and DMRs with expression levels of target genes in blood samples and brain samples. Bold indicates FDR significance (i.e., FDR < 0.05). All statistical tests are two-sided.

File Name: Supplementary Data 7
Description: MethReg analysis of matched DNA methylation and gene expression blood sample data from ADNI study. DNAm × TF effect was estimated from the model target gene expression ~ TF activity + DNAm level + DNAm level × TF activity and represents the magnitude of the TF effect on target gene

expression that is modified by DNA methylation. TF effects in low and high DNAm samples were obtained by fitting model target gene expression ~ TF activity in samples with low (or high) methylation levels separately. All analyses were performed after removing covariate effects (age, sex, immune cell-type proportions, batch) in DNA methylation and gene expression data separately. All statistical tests are two-sided.

File Name: Supplementary Data 8
Description: MethReg analysis of matched DNA methylation and gene expression brain samples data from ROSMAP study. DNAm × TF effect was estimated from the model target gene expression ~ TF activity + DNAm level + DNAm level × TF activity and represents the magnitude of the TF effect on target gene expression that is modified by DNA methylation. TF effects in low and high DNAm samples were obtained by fitting model target gene expression ~ TF activity in samples with low (or high) methylation levels separately. All analyses were performed after removing covariate effects (age at death, sex, estimated proportion of neurons, batch) in DNA methylation and gene expression data separately. All statistical tests are two-sided.

File Name: Supplementary Data 9
Description: Results of integrative analysis of DNA methylation and gene expression data using matched brain samples or blood samples. For each tissue, methylation residuals and gene expression residuals were obtained by fitting linear models and extracting residuals to remove effects of age, sex, batch, and cell types. We then performed a principal component analysis to summarize methylation residuals at significant CpGs by the first PC (PC1), which is a weighted linear combination of the covariate-adjusted methylation values at significant CpGs; these are the methylation PC scores. Next, we tested the methylation PC scores (i.e., PC1) against genome-wide gene expression residuals using linear model $\log_2$(gene expression) ~ PC1. For significant CpGs, in blood sample analysis, we considered the top 50 most significant AD-associated CpGs with *P*-value < 10^{-5} in the current blood samples meta-analysis. For brain sample analysis, we considered the 3751 CpGs with FDR < 0.05 in our previous meta-analysis of brain sample meta-analysis (PMID: 33257653). All statistical tests are two-sided.

File Name: Supplementary Data 10
Description: GSEA analysis results of Canonical pathways (MSigDB C2:CP gene sets) in the integrative methylation and gene expression data using brain or blood samples. For each tissue, methylation residuals and gene expression residuals were obtained by fitting linear models and extracting residuals to remove effects of age, sex, batch, and cell types. We then performed a principal component analysis to summarize methylation residuals at significant CpGs by the first PC (PC1), which is a weighted linear combination of methylation residuals at the significant CpGs. These are the methylation PC scores. Next, we tested the methylation PC scores (i.e., PC1) against genome-wide gene expression residuals using a linear model $\log_2$(gene expression) ~ methylation PC score. For blood sample analysis, we used matched methylation-gene expression data from the ADNI study and considered the top 50 most significant AD-associated CpGs with *P*-value < 10^{-5} in the current blood samples meta-analysis. For brain sample analysis, we used matched methylation-gene expression data from the ROSMAP study and considered the 3751 CpGs with FDR < 0.05 in our previous meta-analysis of brain samples meta-analysis (PMID: 33257653). The absolute value of gene-wise t-statistics for methylation PC scores obtained from the linear models was next used as input for GSEA analysis. Leading edge genes are the core group of genes that accounts for the pathway association signal. The Jaccard index estimates the proportion of overlapping genes in two sets of genes. All statistical tests are two-sided.

File Name: Supplementary Data 11
Description: GSEA analysis of Gene Ontology terms (MSigDB C5:BP gene sets) in the integrative analysis of methylation and gene expression data using brain samples or blood samples. For each tissue, methylation

residuals and gene expression residuals were obtained by fitting linear models and extracting residuals to remove effects of age, sex, batch, and cell types. We then performed a principal component analysis to summarize methylation residuals at the significant CpGs by the first PC (PC1), which is a weighted linear combination of methylation residuals at the significant CpGs. These are the methylation PC scores. Next, we tested the methylation PC scores (i.e., PC1) against genome-wide gene expression residuals using a linear model $\log_2$(gene expression)) ~ methylation PC score. For blood samples analysis, we used matched methylation-gene expression data from the ADNI study and considered the top 50 most significant AD-associated CpGs with P-value $< 10^{-5}$ in the current blood samples meta-analysis. For brain samples analysis, we used matched methylation-gene expression data from the ROSMAP study and considered the 3751 CpGs with FDR < 0.05 in our previous meta-analysis of brain samples meta-analysis (PMID: 33257653). The absolute value of gene-wise t-statistics for methylation PC scores obtained from the linear models was next used as input for GSEA analysis. Leading edge genes are the core group of genes that accounts for the pathway association signal. The Jaccard index estimates the proportion of overlapping genes in two sets of genes. All statistical tests are two-sided.

File Name: Supplementary Data 12
Description: A total of 72 CpG - mQTL pairs were significant in both brain and blood sample analyses. The blood mQTLs and brain mQTLs were obtained from the GoDMC database and xQTL server, respectively. Definitions for columns under "Blood mQTLs" can be obtained from the README file at http://mqtldb.godmc.org.uk/downloads. All statistical tests are two-sided.

File Name: Supplementary Data 13
Description: A total of 3045 blood mQTLs overlapped with the 24 GWAS nominated LD blocks in Kunkle et al. (2019) (PMID: 30820047). The mQTLs in blood were obtained from the GoDMC database. Annotations for CpGs include the location of the CpG based on hg19/GRCh37 genomic annotation (Chr, Position), nearby genes based on GREAT annotation and Illumina gene annotation (UCSC_RefGene_Name), the type of associated genomic feature (UCSC_RefGene_Group), and location with respect to CpG islands (Relation_to_Island).

File Name: Supplementary Data 14
Description: Three AD-associated CpGs overlapped with AD GWAS loci (LDblockGRCh37) reported in Kunkle et al. (2019). Annotations for CpGs include the location of the CpG based on hg19/GRCh37 genomic annotation (Chr, Position), nearby genes based on GREAT annotation and Illumina gene annotation (UCSC_RefGene_Name), the type of associated genomic feature (UCSC_RefGene_Group), and location with respect to CpG islands (Relation_to_Island).

File Name: Supplementary Data 15
Description: Quality control (QC) information on pre-processing of DNA methylation samples and probes.

File Name: Supplementary Data 16
Description: Results of internal validation that compared logistic regression models with or without APOE effect. A 10-fold cross-validation using the ADNI dataset showed the estimated average AUCs for the best performing logistic regression models with and without *APOE* status were 0.810 and 0.691, respectively. The MRS was computed as the sum of methylation beta values (for prioritized CpGs in cross-tissue analysis) weighted by their estimated effect sizes obtained in the meta-analysis.
